# Comparative Physiological and Biochemical Changes in Tomato (*Solanum lycopersicum* L.) under Salt Stress and Recovery: Role of Antioxidant Defense and Glyoxalase Systems

**DOI:** 10.3390/antiox8090350

**Published:** 2019-09-01

**Authors:** Khursheda Parvin, Mirza Hasanuzzaman, M. H. M. Borhannuddin Bhuyan, Kamrun Nahar, Sayed Mohammad Mohsin, Masayuki Fujita

**Affiliations:** 1 Laboratory of Plant Stress Responses, Department of Applied Biological Sciences, Faculty of Agriculture, Kagawa University, Miki-Cho, Kita-Gun, Kagawa 761-0795, Japan; 2 Department of Agronomy, Sher-e-Bangla Agricultural University, Sher-e-Bangla Nagar, Dhaka 1207, Bangladesh; 3 Department of Agricultural Botany, Sher-e-Bangla Agricultural University, Sher-e-Bangla Nagar, Dhaka 1207, Bangladesh

**Keywords:** antioxidant defense, methylglyoxal, oxidative stress, post stress restoration, reactive oxygen species

## Abstract

Salinity toxicity and the post-stress restorative process were examined to identify the salt tolerance mechanism in tomato, with a focus on the antioxidant defense and glyoxalase systems. Hydroponically grown 15 day-old tomato plants (*Solanum lycopersicum* L. cv. Pusa Ruby) were treated with 150 and 250 mM NaCl for 4 days and subsequently grown in nutrient solution for a further 2 days to observe the post-stress responses. Under saline conditions, plants showed osmotic stress responses that included low leaf relative water content and high proline content. Salinity induced oxidative stress by the over-accumulation of reactive oxygen species (H_2_O_2_ and O_2_^•−^) and methylglyoxal. Salinity also impaired the non-enzymatic and enzymatic components of the antioxidant defense system. On the other hand, excessive Na^+^ uptake induced ionic stress which resulted in a lower content of other minerals (K^+^, Ca^2+^, and Mg^2+^), and a reduction in photosynthetic pigment synthesis and plant growth. After 2 days in the normal nutrient solution, the plants showed improvements in antioxidant and glyoxalase system activities, followed by improvements in plant growth, water balance, and chlorophyll synthesis. The antioxidant and glyoxalase systems worked in concert to scavenge toxic reactive oxygen species (ROS), thereby reducing lipid peroxidation and membrane damage. Taken together, these findings indicate that tomato plants can tolerate salinity and show rapid post-stress recovery by enhancement of their antioxidant defense and glyoxalase systems.

## 1. Introduction

Salinity is an abiotic stress factor that drastically hinders plant growth and productivity by altering plant cellular homeostasis, physiology, metabolism, and biochemistry through the imposition of osmotic and ionic stress [1]. The first effect of a high concentration of salt in a plant is osmotic stress. Under this condition, the plant undergoes osmotic adjustment, reduces cell expansion and cell division, induces stomatal closure, and decreases its leaf area, thereby suppressing photosynthesis and growth [2]. Plants under salt stress accumulate high amounts of sodium ion (Na^+^) and leak large quantities of potassium ion (K^+^) from their cells through enhanced Na^+^/K^+^ exchange, which leads to ionic stress [2]. Besides, high Na^+^ uptake causes chlorosis and necrosis of mature leaves, along with premature senescence, through disruption of protein synthesis and enzyme activity [2,3].

Under natural conditions, a plant responds and adapts to salinity through alterations in cellular metabolism and defense mechanisms [1,3,4]. Different abiotic stresses, including salinity, enhance the production of reactive oxygen species (ROS), such as singlet oxygen (^1^O_2_), superoxide (O_2_^•−^) anion, hydrogen peroxide (H_2_O_2_), and hydroxyl radical (OH**^•^**), thereby inducing oxidative stress [5,6]. Oxidative stress damages the components of cellular organelles, such as lipids, nucleic acids, and proteins, which in turn disrupts normal cell metabolism and membrane functions, while triggering lipid peroxidation and, ultimately, programmed cell death [7]. Thus, control of ROS production is essential not only to prevent injurious ROS effects but also to ensure proper execution of their signaling functions [8].

Plants have developed several defense mechanisms, as well as signaling actions, to regulate both the formation and the removal of ROS to avoid oxidative damage [8,9]. The antioxidant defense system efficiently scavenges excess ROS by the coordinated action of different enzymes, that include superoxide dismutase (SOD), catalase (CAT), ascorbate peroxidase (APX), monodehydroascorbate reductase (MDHAR), dehydroascorbate reductase (DHAR), glutathione reductase (GR), glutathione peroxidase (GPX), and glutathione *S*-transferase (GST), as well as by the involvement of multiple non-enzymatic reactions [6,10].

One less well-studied compound with the ability to generate ROS and oxidative stress in plants is methylglyoxal (MG), another cytotoxic and highly reactive compound. However, plants have a glyoxalase system, consisting of the glyoxalase I (Gly I) and glyoxalase II (Gly II) enzymes that, together with glutathione (GSH), detoxify MG into nontoxic compounds [5]. This raises the possibility that an efficient concomitant regulation of the antioxidant defense and glyoxalase systems may be important in conferring salt tolerance in plants.

Salt stress has detrimental effects on plants, but the plant response during the post-stress period is also very critical for recovery from stress-induced damage and subsequent survival. As in the stress condition, plants also show responsive behaviors when the stress is relieved that allow recovery from salt-induced injury. One previous report has described the restoration of growth in salt-stressed plants upon removal of the stress [11]. Other reports have indicated a reduction in oxidative damage and toxic ion accumulations [12]. However, information is lacking regarding the physiological responses and the involvement of the antioxidant and glyoxalase systems after the withdrawal of salt stress. This gap in knowledge prompted the present study, which focused on the plant physiological attributes, as well as the activities of the antioxidant defense and glyoxalase systems, during a post-stress period to obtain a better understanding of the mechanisms that underlie the recovery of plants from salt stress.

## 2. Materials and Methods

### 2.1. Plant Materials and Stress Treatments

Uniform and healthy tomato (*Solanum lycopersicum* L. cv. Pusa Ruby) seeds were surface sterilized with 70% ethanol (5 min). Fifteen seeds were placed on two layers of moistened filter paper in Petri plates and incubated in a germination chamber. After 5 d, the number of plants per plate was reduced to 10 healthy plants and the plates were transferred to a growth chamber. The plants were supplied with full strength Hoagland nutrient solution [13] and grown under controlled conditions (light: 350 μmol photon m^−1^ s^−2^, photoperiod: 16/8 h of light/dark, temperature: 25 ± 2 °C, and relative humidity: 65–70%) for the next 10 d. Several trials were conducted prior to the actual experiment to determine the highest salt level exposure with the shortest recovery period. We found that tomato plants recovered within 48 h from the damage induced by 250 mM NaCl. Therefore, we selected 150 and 250 mM NaCl and 96 h as the stress conditions and a subsequent 48 h in normal nutrient solution for the recovery condition to investigate the recovery mechanism. After 96 h of salt stress, the plants were moved to the recovery solution by removing the salt solution, washing the plants with distilled water, and then supplying the nutrient solution. The third and fourth leaves of the tomato plants were analyzed after both the stress and recovery phases. The whole experiment was conducted three times and included three replications per treatment, with 10 plants per replication. Morphological data were obtained as the averaged values from 10 randomly selected plants.

### 2.2. Determination of Growth Parameters

Plant height, root length, number of leaves, and stem girth were recorded. The shoots, leaves, and roots were removed and weighed to determine fresh weights and were then dried at 80 °C for 48 h to obtain dry weights.

### 2.3. Determination of Na and Other Mineral Nutrients

Sodium, K, Ca, and Mg content in the plant shoots and roots were determined from oven dried material (72 h; 70 °C) digested with an acid mixture (HNO_3_:HClO_4_; 5:1, *v*/*v*) at 70 °C for 48 h [2]. The mineral contents were analyzed by atomic absorption spectrophotometry (AA-7000 instrument, Shimadzu, Japan).

### 2.4. Measurement of Photosynthetic Pigment Contents

Leaf chlorophyll *a* (Chl *a*), chlorophyll *b* (Chl *b*), and carotenoid (Car) contents were measured according to Wellburn [14] using absolute ethanol.

### 2.5. Measurement of Relative Water and Free Proline Content in Leaves

Relative water content (RWC) was determined from fully developed leaves following the protocol of Barrs and Weatherley [15]. The fresh weight (FW) of 10 leaves was measured, the leaves were then immersed in distilled water, and turgid weight (TW) was obtained 8 h later. Dry weight (DW) was measured after drying the leaf samples at 80 °C for 48 h. The RWC was calculated using following equation:

RWC (%) = (FW − DW)/(TW − DW) × 100(1)

Free proline (Pro) content in the leaf tissue was determined according to Bates et al. [16] using acid ninhydrin prepared with glacial acetic acid and phosphoric acid. The ninhydrin-Pro complex was extracted with toluene.

### 2.6. Evaluation of Oxidative Stress Markers

Lipid peroxidation was measured as the malondialdehyde (MDA) content based on the production of thiobarbituric acid reactive substances (TBARS) [17]. The H_2_O_2_ levels were measured according to Hossain et al. [18] using potassium iodide (KI).

Increased generation of H_2_O_2_ and O_2_^•−^ in tomato leaves was histochemically confirmed using 3,3-diaminobenzidine (DAB) and nitrobluetetrazolium (NBT), respectively [19].

Electrolyte leakage (EL) from leaf and root tissues was estimated following the protocol of Dionisio-Sese and Tobita [20].

### 2.7. Protein Quantification and Enzyme Activity Assays

Fresh leaf tissue (0.5 g) was homogenized and extracted according to Hasanuzzaman et al. [21], which was then used to analyze protein content and the enzyme activity assay.

The protein content was determined according to Bradford [22] using bovine serum albumin as a protein standard.

Lipoxygenase (LOX; EC: 1.13.11.12) activity was determined following Doderer et al. [23]. SOD (EC: 1.15.1.1) activity was quantified with a xanthine–xanthine oxidase system [4]. CAT (EC: 1.11.1.6), GR (EC: 1.6.4.2), and GST (EC: 2.5.1.18) activities were measured using the protocol of Hasanuzzaman et al. [21]. APX (EC: 1.11.1.11) activity was evaluated according to the method of Nakano and Asada [24], whereas MDHAR (EC: 1.6.5.4), DHAR (EC: 1.8.5.1), and GPX (EC: 1.11.1.9) activities were measured as described by Nahar et al. [7]. Gly I (EC: 4.4.1.5) and Gly II (EC: 3.1.2.6) activities were assayed according to Hasanuzzaman et al. [21] and Principato et al. [25], respectively.

### 2.8. Determination of Ascorbate and Glutathione Content

Fresh leaf tissue (0.5 g) was homogenized with 3 mL 5% trichloroacetic acid (TCA) [18]. After centrifugation (11,500× *g*) for 15 min at 4 °C, the collected supernatant was used for determination of ascorbate (AsA), DHA (dehydroascorbate), GSH, and oxidized glutathione (GSSG) pool sizes using the protocols of Parvin et al. [3].

### 2.9. Determination of Methylglyoxal Content

Methylglyoxal (MG) content was measured as described by Nahar et al. [7]. Leaves were first homogenized with 5% perchloric acid (PCA) and centrifuged at 11,000× *g*. The supernatant was read at 288 nm spectrophotometrically after adding *N*-acetyl-l-cysteine.

### 2.10. Statistical Analysis

The measured data were statistically analyzed using XLSTAT 2018 software [26]; three replications were used for analysis of variance (ANOVA). The mean differences were compared using Fisher’s least significant difference (LSD) test at the 5% level of significance.

## 3. Results

### 3.1. Growth Restoration

**Figure 1:**
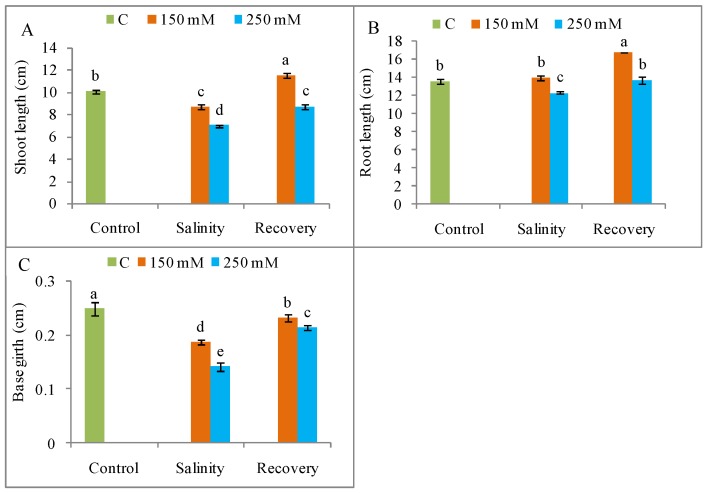
Tomato plant growth (**A**) shoot height, (**B**) root length, and (**C**) base girth under salinity (150 and 250 mM NaCl) and after a 48 h recovery period. Data means (±SD) were calculated from three replications. Statistically significant values are indicated by dissimilar letters (Fisher’s least significant difference (LSD) test, *P* ≤ 0.05).

**Figure 2:**
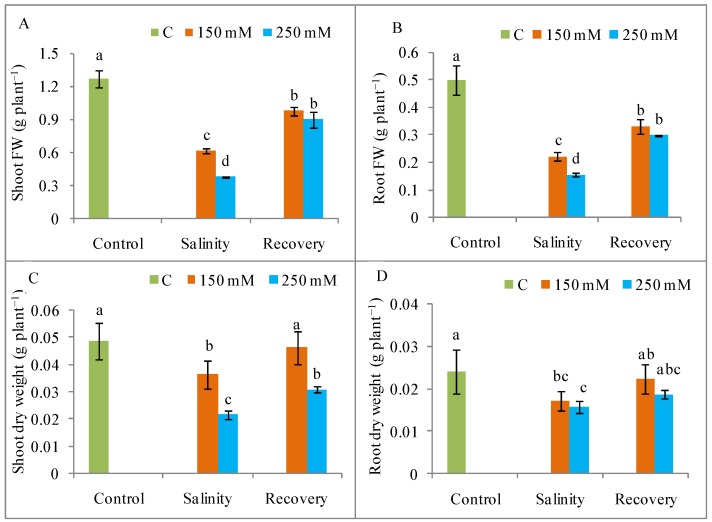
Plant biomass content (**A**) shoot fresh weight, (**B**) root fresh weight, (**C**) shoot dry weight, and (**D**) root dry weight in tomato plants under salinity (150 and 250 mM NaCl) and after a 48 h recovery period. Data means (±SD) were calculated from three replications. Statistically significant values are indicated by dissimilar letters (Fisher’s LSD test, *P* ≤ 0.05).

**Figure 3:**
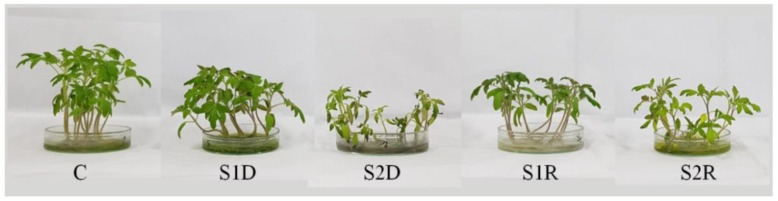
Visual differences of tomato plants under salinity (150 and 250 mM NaCl) and after a 48 h recovery period. (C, control; S1D, 150 mM NaCl; S2D, 250 mM NaCl; S1R, recovered 150 mM NaCl; and S2R, recovered 250 mM NaCl).

Salinity suppressed plant growth and dry matter accumulation, with a maximum decrease in shoot and root length, stem girth, and shoot and root FW and DW observed in the plants treated with 250 mM salt compared to unstressed control plants; seedling growth was restored after the 48 h recovery period (Figure 1 and Figure 2). The phenotypic appearance of the plants also indicated a reduction in growth and vigor under salt stress in relation to control plants, but a normal phenotype was restored after the recovery period (Figure 3).

### 3.2. Na^+^ Ion Homeostasis and Mineral Nutrition

**Figure 4:**
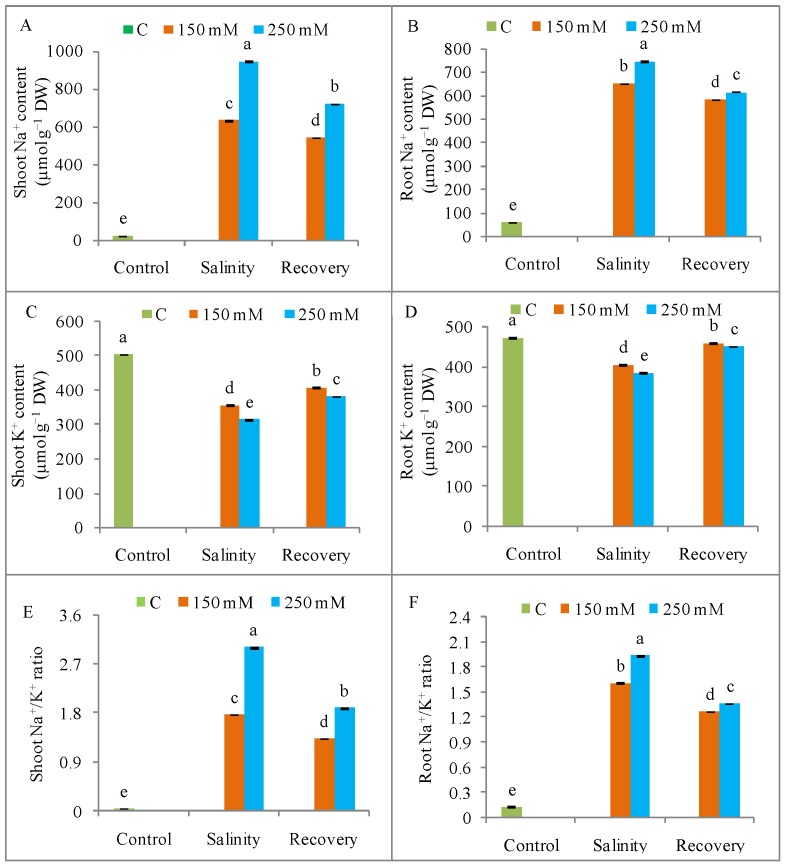
Content of Na^+^, K^+^ and Na^+^/K^+^ ratio (**A**) shoot Na^+^, (**B**) root Na^+^, (**C**) Shoot K^+^, (**D**) Root K^+^, (**E**) Shoot Na^+^/K^+^ ratio, and (**F**) root Na^+^/K^+^ in tomato plants under salinity (150 and 250 mM NaCl) and after a 48 h recovery period. Data means (±SD) were calculated from three replications. Statistically significant values are indicated by dissimilar letters (Fisher’s LSD test, *P* ≤ 0.05).

**Figure 5:**
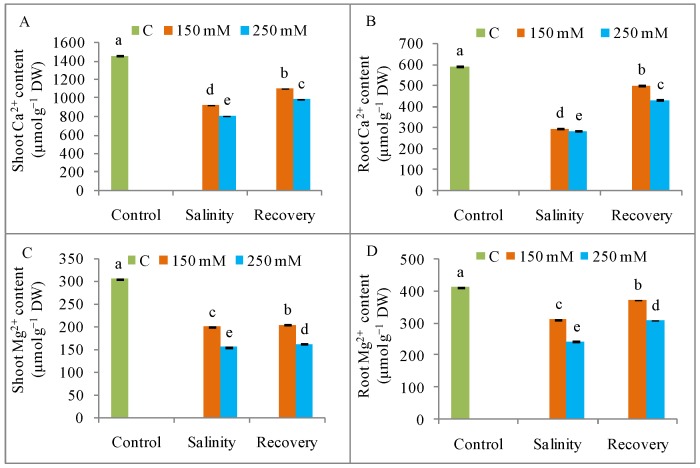
Content of Ca^2+^ and Mg^2+^ (**A**) shoot Ca^2+^, (**B**) root Ca^2+^, (**C**) shoot Mg^2+^, and (**D**) root Mg^2+^ in tomato plants under salinity (150 and 250 mM NaCl) and after a 48 h recovery period. Data means (±SD) were calculated from three replications. Statistically significant values are indicated by dissimilar letters (Fisher’s LSD test, *P* ≤ 0.05).

Salinity induced a higher Na^+^ uptake, along with a lowered K^+^, Ca^2+^, and Mg^2+^ content, in both shoots and roots when compared to unstressed control plants. Compared to control, shoot Na^+^ accumulation increased by as much as 40-fold, while root Na^+^ content increased by 12-fold in response to 250 mM NaCl; however, the Na^+^ content in both shoot and root tissues decreased after the 48 h recovery period (Figure 4A,B). The maximum K^+^ loss compared to control, was observed in response to 250 mM NaCl, resulting in an extreme increase in the Na^+^/K^+^ value in both roots and shoots (Figure 4C–F). Exposure to 250 mM NaCl reduced the Ca^2+^ content in shoots and roots by 44% and 49%, respectively; and the Mg^2+^ content by 52% and 41%, respectively compared to unstressed control plants (Figure 5). Mineral homeostasis was restored, with a reduction in Na^+^ and an increase in K^+^, Ca^2+^, and Mg^2+^ contents after the recovery period when compared to the stress condition (Figure 4 and Figure 5).

### 3.3. Photosynthetic Pigment Content

**Figure 6:**
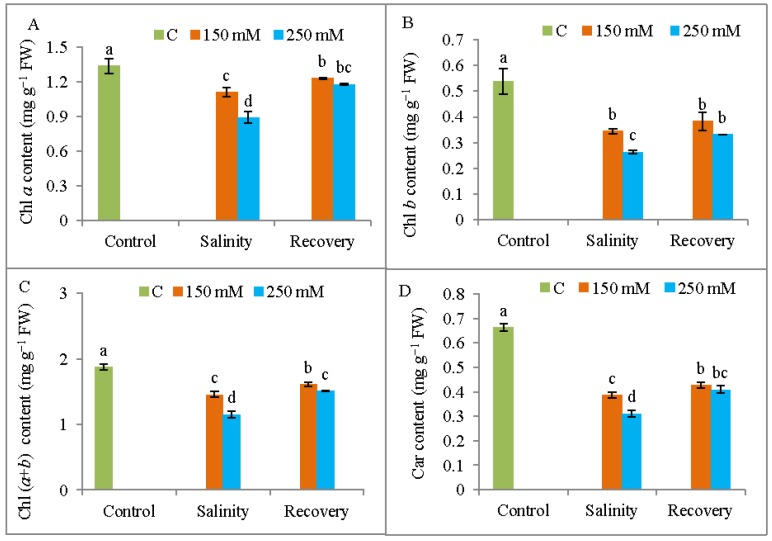
Photosynthetic pigments contents (**A**) chlorophyll *a* (Chl *a*), (**B**) chlorophyll *b* (Chl *b*), (**C**) Chl (*a* + *b*), and (**D**) carotenoid in tomato plants under salinity (150 and 250 mM NaCl) and after a 48 h recovery period. Data means (±SD) were calculated from three replications. Statistically significant values are indicated by dissimilar letters (Fisher’s LSD test, *P* ≤ 0.05).

The leaf contents of Chl *a* and Chl *b* decreased by 33% and 51%, respectively, in response to 250 mM NaCl compare to control plants. The Chl (*a* + *b*) content was therefore similarly reduced relative to the unstressed control plants. However, both Chl *a* and Chl *b* contents were increased respective to the relevant stress treatments after the recovery period (Figure 6A–C). Car content also decreased by 42% and 53% in response to 150 and 250 mM NaCl, respectively, and it increased after the recovery period (Figure 6D).

### 3.4. Osmotic Adjustment and RWC

**Figure 7:**
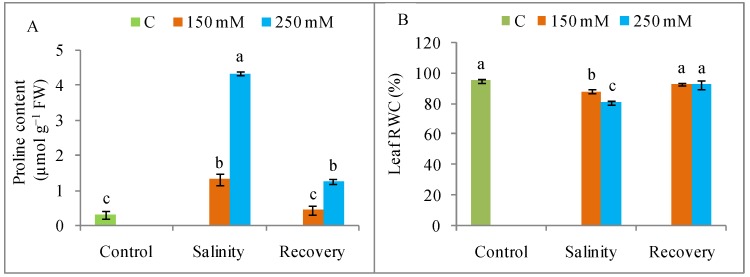
Osmotic status (**A**) proline (Pro) content, (**B**) leaf relative water content (RWC) in tomato plants under salinity (150 and 250 mM NaCl) and after a 48 h recovery period. Data means (±SD) were calculated from three replications. Statistically significant values are indicated by dissimilar letters (Fisher’s LSD test, *P* ≤ 0.05).

Proline levels were greatly increased by salt exposure compared to control (Figure 7A), but these levels declined significantly after the recovery period, by 68% and 72% in the plants treated with 150 and 250 mM NaCl, respectively. Salt stress reduced the leaf RWC, but this was restored after the recovery period (Figure 7B).

### 3.5. Oxidative Stress

**Figure 8:**
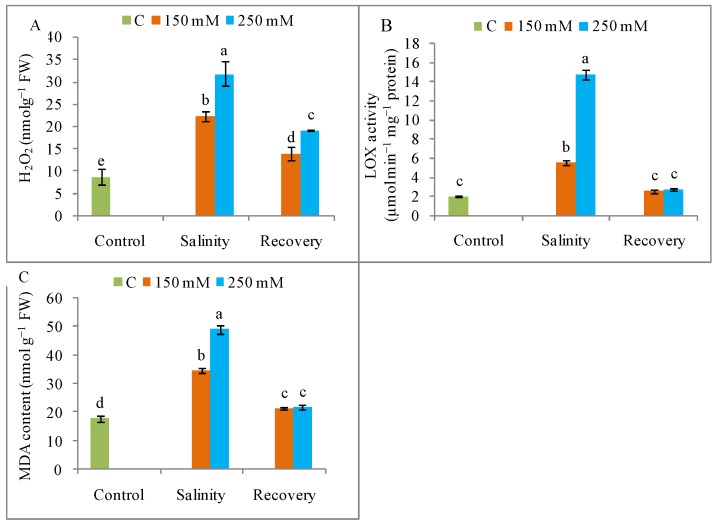
Oxidative stress markers (**A**) H_2_O_2_ content, (**B**) lipoxygenase (LOX) activity, (**C**) malondialdehyde (MDA) content in tomato plants under salinity (150 and 250 mM NaCl) and after a 48 h recovery period. Data means (±SD) were calculated from three replications. Statistically significant values are indicated by dissimilar letters (Fisher’s LSD test, *P* ≤ 0.05).

**Figure 9:**
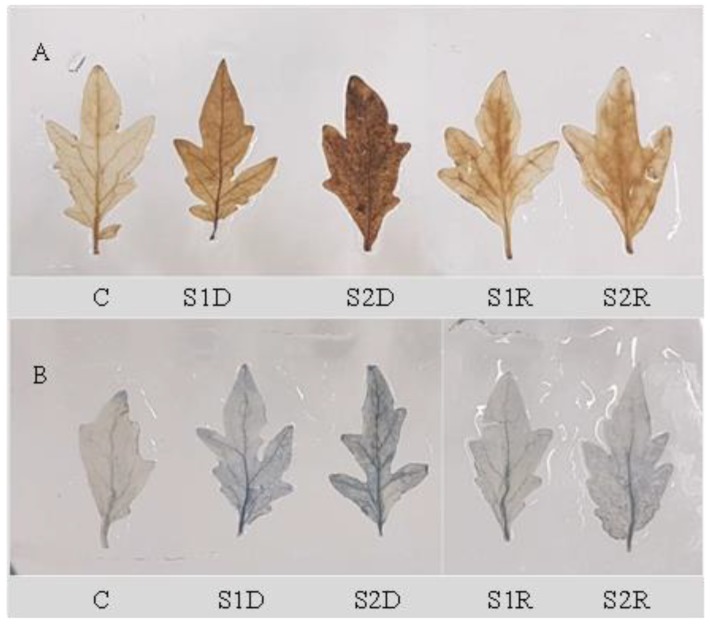
Histochemical detection of oxidative stress (**A**, H_2_O_2_; **B,** O_2_^•−^) in tomato plants leaves under salinity (150 and 250 mM NaCl) and after a 48 h recovery period. Data means (±SD) were calculated from three replications. Statistically significant values are indicated by dissimilar letters (Fisher’s LSD test, *P* ≤ 0.05).

**Figure 10:**
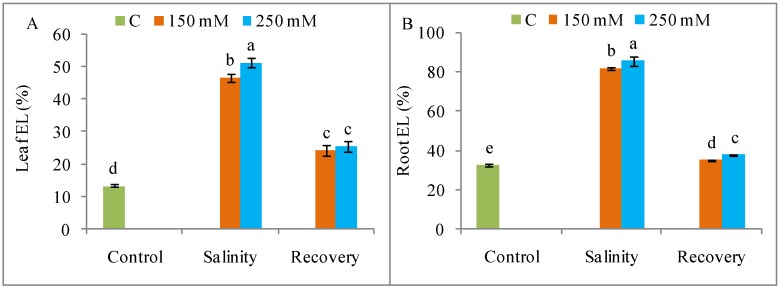
Electrolyte leakage (**A**) leaf, (**B**) root in tomato plants under salinity (150 and 250 mM NaCl) and after a 48 h recovery period. Data means (±SD) were calculated from three replications. Statistically significant values are indicated by dissimilar letters (Fisher’s LSD test, *P* ≤ 0.05).

The H_2_O_2_ production was higher under NaCl stress than in the unstressed control condition (Figure 8A), as also revealed by histochemical staining (Figure 9). The generation of ROS was lower after the recovery period than with stress alone (Figure 8A and Figure 9). As documented in Figure 8B, the salt treatments (150 and 250 mM) also stimulated the activity of LOX, but this activity declined to control levels after the recovery period (Figure 8A,B). Compared to control, lipid peroxidation was increased by salt stress but returned to control levels after the recovery period (Figure 8C). Salt stress caused a higher and dose-dependent electrolyte leakage (EL) from both roots and leaves compared to unstressed plants, and this leakage was also reduced after the recovery period (Figure 10).

### 3.6. Antioxidant Enzyme Activities

**Figure 11:**
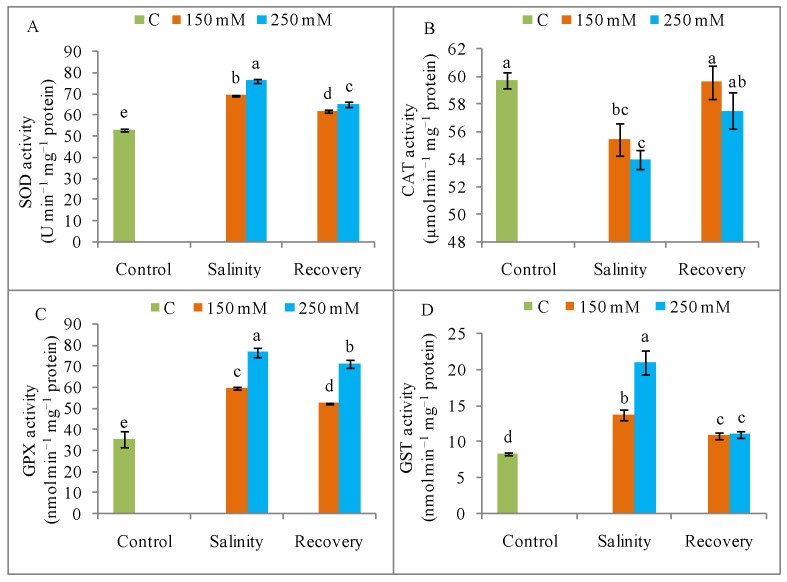
(**A**) Activities of superoxide dismutase (SOD), (**B**) catalase (CAT), (**C**) glutathione peroxidase (GPX), and (**D**) glutathione *S*-transferase (GST) in tomato plants under salinity (150 and 250 mM NaCl) and after a 48 h recovery period. Data means (±SD) were calculated from three replications. Statistically significant values are indicated by dissimilar letters (Fisher’s LSD test, *P* ≤ 0.05).

When compared with unstressed control plants, the SOD activity was increased by 30% and 43% after treatment with 150 and 250 mM NaCl, respectively (Figure 11A), whereas SOD activity decreased after the recovery period. Compared to control, CAT activity was decreased by salt stress but was restored to the control level after the recovery period (Figure 11B).

**Figure 12:**
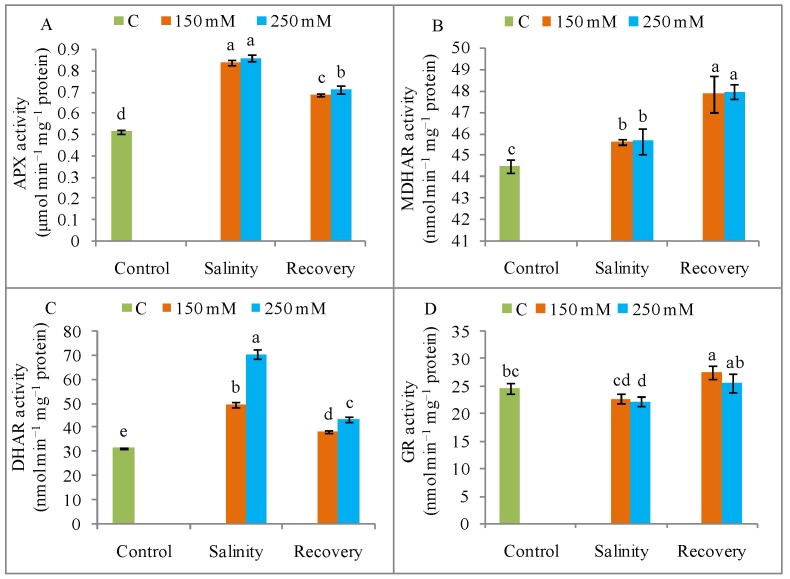
Activities of ascorbate peroxidase (APX) (**A**), monodehydroascorbate reductase (MDHAR) (**B**), dehydroascorbate reductase (DHAR) (**C**), and glutathione reductase (GR) (**D**) in tomato plants under salinity (150 and 250 mM NaCl) and after a 48 h recovery period. Data means (±SD) were calculated from three replications. Statistically significant values are indicated by dissimilar letters (Fisher’s LSD test, *P* ≤ 0.05).

The APX activity was significantly increased by salinity (250 mM NaCl) when compared to the unstressed control condition, but this activity declined after the recovery period (Figure 12A). The MDHAR activity was increased by salt stress compared to control, but its activity doubled after the recovery period (Figure 12B). Compared to control, the DHAR activity increased noticeably under salt stress (Figure 12B), but declined again after the recovery period (Figure 12C). The GR activity was slightly decreased by salt exposure compared with the unstressed condition, but increased after the recovery period (Figure 12D). The thiol-dependent enzymes, GPX and GST, showed increased activity in response to NaCl stress compared to control, but both these activities were reduced after the recovery period (Figure 11C,D).

### 3.7. Non-Enzymatic Antioxidant Levels

**Figure 13:**
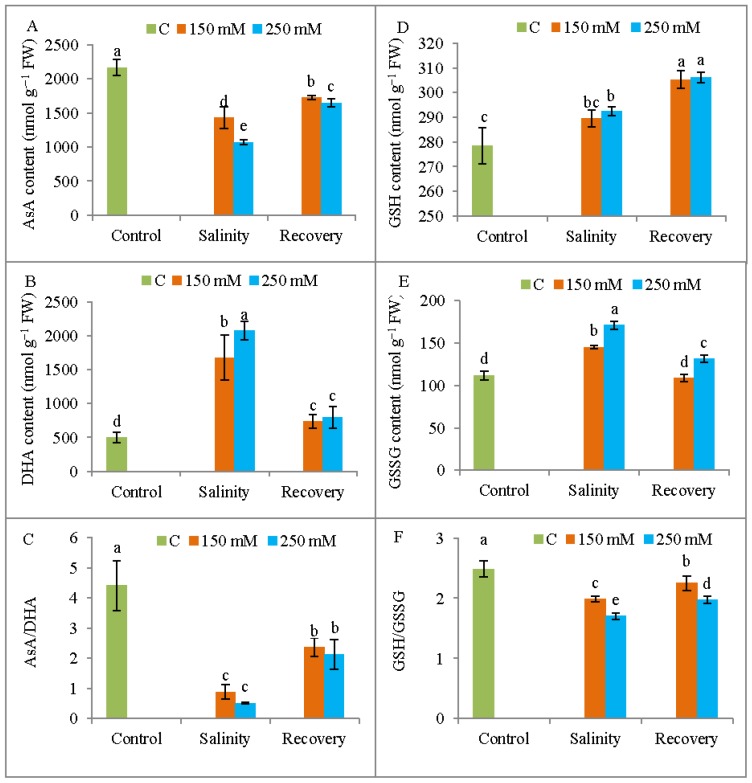
Content of AsA (**A**), dehydroascorbate (DHA) (**B**), AsA/DHA ratio (**C**), GSH (**D**), GSSG(**E**), and GSH/GSSH ratio (**F**) in tomato plants under salinity (150 and 250 mM NaCl) and after a 48 h recovery period. Data means (±SD) were calculated from three replications. Statistically significant values are indicated by dissimilar letters (Fisher’s LSD test, *P* ≤ 0.05).

A significant reduction in AsA content and a significant increase in DHA content were observed in response to both 150 and 250 mM NaCl treatments when compared to the unstressed control plants, but these levels approached control levels after the recovery period (Figure 13A,B). Compared to control, the GSH content increased in response to salinity stress and continued to increase after the recovery period (Figure 13D). The GSSG content was increased by salinity stress in comparison with control, but the levels declined after the recovery period (Figure 13E). Salt stress reduced the redox ratio of AsA/DHA and GSH/GSSG compared with unstressed ones, but these ratios were again increased after the recovery period (Figure 13C,F).

### 3.8. MG Detoxification

**Figure 14:**
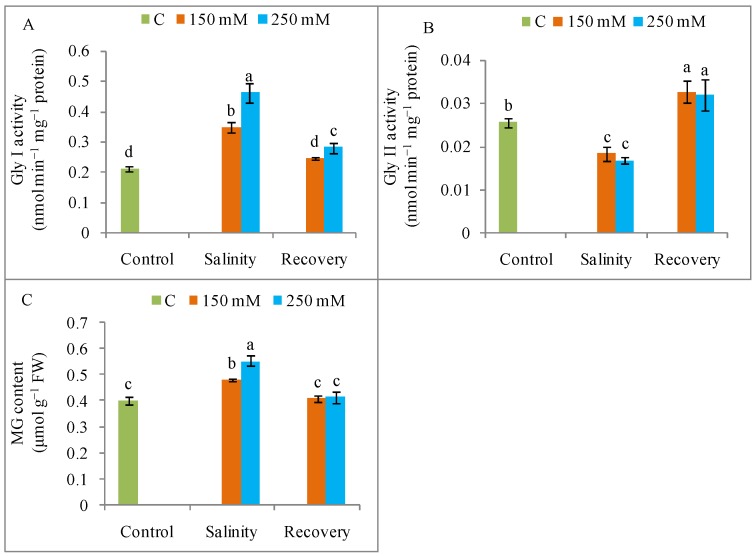
Methylglyoxalase detoxification (**A**) and activities of glyoxalase enzymes (**B**) Gly I, (**C**) Gly II in tomato plants under salinity (150 and 250 mM NaCl) and after a 48 h recovery period. Data means (±SD) were calculated from three replications. Statistically significant values are indicated by dissimilar letters (Fisher’s LSD test, *P* ≤ 0.05).

Salinity increased Gly I and decreased Gly II activity, while increasing the MG content in the salt-stressed plants compared to the unstressed control plants (Figure 14). Compared to stressed conditions, the Gly I activity declined, the Gly II activity increased, and the MG content decreased during the recovery period (Figure 14).

## 4. Discussion

Salt stress drastically inhibits seedling growth and biomass accumulation [3]. Previous reports by Manai et al. [27] and Martinez et al. [28] showed a reduction in the FW of tomato shoots and roots, as well as a decrease in root length, in response to salinity. The reason for salinity-induced growth restriction might be a salt-mediated reduction in cell growth [29]. In the present study, tomato plants showed a significant restoration of normal growth after the recovery period. A similar recovery of growth, indicated by increases in leaf area and shoot DW, has been previously reported for salt-stressed *Vigna unguiculata* [30]. Similarly, Acosta-Motos et al. [31] found that a recovery period after salinity stress restored growth in *Eugenia myrtifolia* L., in agreement with our findings. Under field conditions, salt stress recovery often happens due to normal irrigation or rainfall and growth restoration upon recovery implies a significant improvement in salinity tolerance after removal of a salt stress.

The reduced growth due to salinity stress indicates a need to study ion accumulation in plant cells. In the present case, tomato plants suffered from excess Na^+^ accumulation and a consequent K^+^ loss. Salinity depolarizes the root plasma membrane while activating the outward rectifying K-channels (GORK) of the guard cells, resulting in an increased Na^+^ content and a decreased K^+^ levels [2,32]. The higher Na^+^ content therefore disrupts the Na^+^/K^+^ ratio and ion homeostasis [2,29]. The lower Ca^2+^ content observed in salinity-stressed tomato plants might indicate a replacement of Ca^2+^ by Na^+^. Some reports have suggested that high salinity displaces Ca^2+^ from the cell membrane, resulting in increased membrane permeability and a higher intracellular Na^+^ content [2,7]. Removal of the salt stress significantly decreased the Na^+^ content and the K^+^ content increased, indicating a restoration of ion homeostasis, as evidenced by increasing levels of Ca^2+^ and Mg^2+^; these changes could be responsible for the observed restoration of seedling growth. The study by de Lacerda et al. [11] showed that the lower content of toxic ions in *Sorghum bicolor* seedlings after a salt stress was related to stress tolerance.

Salinity stress also hampers photosynthetic pigment synthesis, resulting in leaf chlorosis, as evident in many previous reports [33,34]. Ahmed et al. [35] and Martinez et al. [28] confirmed the loss of Chl synthesis in salt-stressed plants. The salt-induced reduction in Chl levels might be due to structural damage to the Chl molecules by increased chlorophyllase activity [33]. In the present study, the levels of Chl *a*, Chl *b*, Chl (*a + b*) and Car were restored in the stressed tomato plants after the recovery period, in agreement with previous findings by Acosta-Motos et al. [31]. Therefore, recovery treatments appear to increase plant tolerance and rejuvenate the plants for further growth by restoring Chl synthesis.

Apart from ion toxicity, salinity also causes osmotic stress by altering the water potential in both the growth medium and the plant body. In the present study, tomato plants suffered from osmotic stress indicated by the reduced leaf RWC. High concentrations of NaCl can injure the root system, resulting in lower water uptake [36]. In the present study, salinity increased the Pro levels, which may have increased stress tolerance in the plants by maintaining the osmotic potential, leaf expansion, and stomatal conductance, as well as photosynthesis [7]. Hence, the higher Pro levels observed in the stress-injured plants after the recovery period may indicate an active process for increasing stress tolerance. The recovery period appeared to result in a reduction in osmotic stress due to increased RWC [37].

Tomato plants also showed a quick recovery response from damage induced by high salt exposure, as indicated by the increase in metabolic activities designed to avoid the deleterious effects of short-duration salinity. Salinity increases the generation of ROS (H_2_O_2_, O_2_^•−^) in plants, resulting in oxidative stress. The higher ROS levels cause oxidative damage, including peroxidation of lipid and proteins, pigment destruction, and nucleic acid and DNA damage, and ROS also impairs enzyme activities [28]. Here, a higher H_2_O_2_ content was detected under salt stress, indicating oxidative stress, and the level of this ROS increased with increasing salt concentration, in line with the findings reported separately by Manai et al. [27] and Martinez et al. [28]. Salinity also increases LOX activity, which causes lipid peroxidation. Interestingly, after the recovery period, the oxidative stress was reduced in the tomato plants, as confirmed by the reductions in ROS levels and LOX activity, as well as by the lower MDA content, in agreement with the results reported by Lv et al. [38]. Similarly, Acosta-Motos et al. [31] reported that elevated EL was relieved upon recovery from salinity in *E. myrtifolia*.

The observed restoration of growth in salt-stressed plants and the improvement in osmotic status and mineral homeostasis prompted our exploration of plant antioxidant defense mechanisms. Salt stress increased the SOD activity, which is in agreement with the findings of Ahmad et al. [35]. By contrast, the CAT enzyme works to scavenge toxic H_2_O_2_ [5], and yet the salt-stressed tomato plants showed a reduction in CAT activity under salinity. This finding might indicate a reduced capacity for H_2_O_2_ detoxification, which would result in greater oxidative damage. A similar behavior was reported in tomato by Manai et al. [27]. However, other vital components of this cycle, namely the APX enzyme and AsA, also scavenge toxic H_2_O_2_ and convert it to H_2_O [8]. In the present study, APX activity increased under salinity stress, whereas AsA decreased. The decrease in AsA content was accompanied by a higher DHA content, in agreement with the findings of Ahmad et al. [35], which also reduced AsA content and elevated APX activities in salt-stressed tomato. Acosta-Motos et al. [31] also reported a reduction in AsA content in plants under saline conditions. After a recovery period, the salt-stressed tomato plants showed decreased APX activity and a reduced DHA content, along with a greater AsA content. After the recovery period, the H_2_O_2_ generation had decreased, so less AsA was used to scavenge the ROS and the DHA content declined. The AsA content is modulated by MDHAR and DHAR activities, as well as APX [10], and our salt-stressed tomato plants showed higher activities of MDHAR and DHAR.

Salinity stress also increased the GSH and GSSG content, and reduced the GSH/GSSH ratio, but this ratio increased after the recovery period. Ahmad et al. [35] also reported an increase in GSH content in tomato after salt exposure, and Acosta-Motos et al. [31] also found increased GSH and GSSG contents after a recovery period in salinity-affected plants. In the present study, GR activity was increased in salt-stressed plants. Manai et al. [27] also reported increased GR activity in salt-stressed tomato. Further enhancement of GR activity was observed after the recovery period in saline-treated plants, resulting in further direct modulation of the GSH and GSSG contents and improved plant tolerance. Acosta-Motos et al. [31] showed an increase in GR in salt-treated *E. myrtifolia*, but a reduction in GR activity during a recovery period. Tomato plants in the present study also showed increased activity of GPX and GST under salt stress, but this activity declined after the recovery period, which might be attributed to a decreased H_2_O_2_ content. Previous reports have also shown increased GPX activity in tomato due to salinity [27]. The levels of most of the non-enzymatic and enzymatic antioxidants were increased after the recovery treatment, indicating an enhanced salt tolerance.

Higher MG content in the salt-treated plants was due to the lower activities of glyoxalase enzymes, but these responses were reversed after the stress recovery. Gly I activity was highly involved in MG detoxification under salinity and the concomitant decrease in lowered GSH content. But, upon recovery, Gly II activity increased while Gly I activity decreased thus reducing MG generation, which might have contributed to a higher content of GSH. Therefore, the glyoxalase system was also stimulated during the recovery period in salt-stressed plants, thereby contributing to higher GSH content for controlling ROS and better salt tolerance in tomato plants.

## 5. Conclusions

Salt-stressed tomato plants recover very quickly at the seedling stage by invoking their efficient antioxidant defense and glyoxalase systems. Salt stress restricted seedling growth by imposing oxidative, ionic, and osmotic stresses, along with suppression of these plant defense mechanisms. The recovery period allowed the removal of the salt-induced ionic toxicity by reducing the Na^+^ accumulation and increasing K^+^, Ca^2+^, and Mg^2+^ contents in both the shoots and roots. The recovery treatment also allowed restoration of the photosynthetic pigment levels in salt-injured plant leaves, which correlated with better seedling growth. The Pro content was reduced, concomitantly with higher RWC, after the recovery period, thereby confirming the alleviation of salt-induced osmotic stress. During the recovery period, both non-enzymatic and enzymatic antioxidants, along with glyoxalase enzymes, were apparently able to detoxify the salt-induced ROS. Interestingly, Gly II, rather than Gly 1, showed a pronounced involvement in MG detoxification in the recovered seedling. The positive recovery of tomato plants from the toxic effects of a saline stress, as observed in the present study, indicates a need for further in-depth studies that include phytohormone signaling and crosstalk responses.
